# Genomic Sequence of a Czech Isolate of Erysimum Latent Virus from *Sisymbrium altissimum*

**DOI:** 10.3390/plants13182554

**Published:** 2024-09-11

**Authors:** Karima Ben Mansour, Josef Špak, Petr Komínek, Miloslav Zouhar, Pavel Ryšánek, Adrian J. Gibbs

**Affiliations:** 1 Ecology, Diagnostics and Genetic Resources of Agriculturally Important Viruses, Fungi and Phytoplasmas, Crop Research Institute, Drnovská 507, 161 06 Prague, Czech Republic; kominek@vurv.cz; 2 Department of Plant Protection, Faculty of Agrobiology, Food and Natural Resources, Czech University of Life Sciences Prague, Kamýcká 129, 165 00 Prague, Czech Republic; zouhar@af.czu.cz (M.Z.); rysanek@af.czu.cz (P.R.); 3 Czech Academy of Sciences, Biology Centre, Institute of Plant Molecular Biology, Branišovská 31, 370 05 České Budějovice, Czech Republic; spak@umbr.cas.cz; 4 Emeritus Faculty, Australian National University, Canberra 2601, Australia

**Keywords:** Erysimum latent virus, *Sisymbrium altissimum*, HTS, tymovirus

## Abstract

The Erysimum latent virus (ELV), a tymovirus, was first isolated from several wild and cultivated brassicas in Germany. Its virions were shown to be serologically distinct from those of the turnip yellow mosaic virus (TYMV), which is also found in wild and cultivated plants in several European countries but also in other parts of the world. TYMV and ELV were among the first plant viruses to have had their genomes sequenced, and when other tymovirus genomes were sequenced, it was found that, in phylogenies, ELV is probably the basal outlier to all other tymoviruses. Here, we report the near-complete genomic sequence of another isolate of ELV from Czechia. This isolate was found in 1990 in *Sisymbrium altissimum* plants showing mosaic symptoms. It was detected using ELISA tests and electron microscopy. We have now sequenced the full coding sequence of this isolate using contemporary high throughput methods and found that the German and Czech isolates of ELV are closely related and are of the same virus species.

## 1. Introduction

The Erysimum latent virus (ELV) (of species *Tymovirus erysimi*, genus *Tymovirus*, and family *Tymoviridae*) was first isolated from asymptomatic *Erysimum helveticum* in Germany [1]. The virus has since been found in symptomless plants of numerous other wild brassicas, including *E. crepidifolium*, *E. perovskianum*, *E. pulchellum*, and *E. silvestre*, and *Barbarea vulgaris* [2]. In experiments, ELV infects plants belonging to approximately 40 species within the Brassicaceae and several other plant families, and these exhibit symptoms that are similar to those caused by the turnip yellow mosaic virus (TYMV). No serological cross-reaction was detected between the virions of ELV and TYMV [3], and limited gene sequencing found ELV to be genetically distinct from all other tymoviruses, including the eggplant mosaic virus (EMV), Ononis yellow mosaic virus (OYMV), and Kennedya yellow mosaic virus (KYMV) [3,4]. Flea beetles, four species of the genus *Phyllotreta*, were found to transmit the virus [5].

ELV was also found, using ELISA serological tests and electron microscopy [6], in Czechia in 1990 in *Sisymbrium altissimum* plants showing mosaic symptoms. Špak et al. (1993) compared the two ELV isolates (ELV-Cz and ELV-De) in host range tests and found that *Brassica pekinesis* was resistant to the Czech isolate (ELV-Cz), whereas *Raphanus sativus* was more susceptible to the Czech isolate than to the German isolate (ELV-De) [6].

The Genbank database only contains the genomic sequence of ELV-De [3,4], and this was determined using Sanger sequencing in the early days of genome sequencing; the isolate was found to be a basal outlier to all other tymoviruses based on phylogenetic analyses [4]. Therefore, given the outlier status of that sequence, and hence its importance for interpreting the phylogeny of tymoviruses, we used high-throughput sequencing (HTS) to obtain the full coding sequence of ELV-Cz.

## 2. Results and Discussion

HTS sequencing generated approximately 35 million raw reads, which were then trimmed and produced c. 33 million trimmed reads. The reads were filtered to remove reads of the host genome (*Sinapis alba*; accessions for scaffolds, mRNA sequences and chloroplasts, PRJNA214277, NC_045948, and MN176144); then, they were de novo assembled. The BLASTn analysis of the de novo contigs found them to be closest to ELV-De, with an identity of 88.21% nt. Only ELV reads were found, and approximately 31 million trimmed reads were aligned to produce the ELV-Cz contig at 5860 nts long, covering the coding regions. The 5′- and 3′-ends (95 nt and 77 nt, respectively) were predicted using the sequences of other tymoviruses; therefore, the ends required validation by RACE analysis. The mapping of the 31 million trimmed reads against the ELV-Cz sequence resulted in the identification of 30,899,898 reads (92.8%). A search using RDP and 10 other tymovirus genomes found no evidence of recombination.

The resulting ELV sequence (ELV-Cz) was 6031 nucleotides in length. The ELV-Cz sequence was submitted to Genbank, where it was given the accession number PP093825 (BioProject ID: PRJNA1099901).

**Figure 1:**
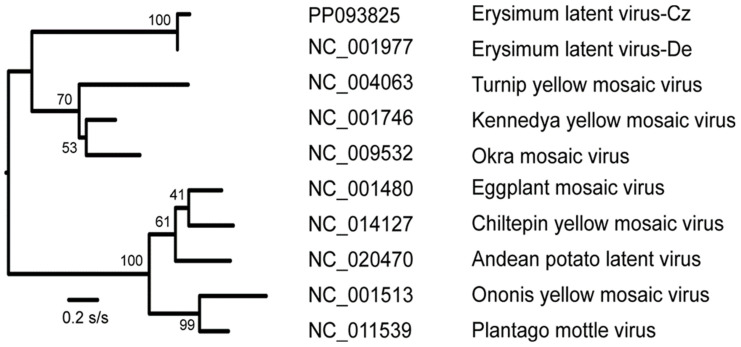
Midpoint-rooted ML phylogenetic tree of 10 CP gene sequences was constructed using the ML facility of MEGAX. The percentage values for nodes were calculated from 500 bootstrap iterations. The scale is 0.2 substitutions/site.

The ELV-Cz sequence has a large cytosine content (33.8%) characteristic of tymoviruses. The NCBI ORF finder identified three open reading frames (ORFs) within the sequence. The longest ORF is 5244 nts and encodes a 1748 aa RNA-dependent RNA polymerase (RdRp). This has a variant of the tymobox (‘GAGTTTGAATTGCTTC’), characteristic of tymoviruses, 21–37 nts from its 3′-terminus, and its last four nts are the first four of the coat protein (CP) genes of 202 aa. [7]. The movement protein (MP) gene overlaps the RdRp gene of the virus. MP initiates seven nts from the 5′ side of the initiation codon of the RdRp gene (-ATGGCCATG-) and encodes a 637 aa movement protein (MP). The CP gene is the most conserved gene of ELV (92.2% nt and 99% aa identity between the two known ELV sequences; Table 1), as well as of other tymoviruses. Figure 1 illustrates an ML phylogeny based on the CP gene of 10 of these viruses.

**Figure 2:**
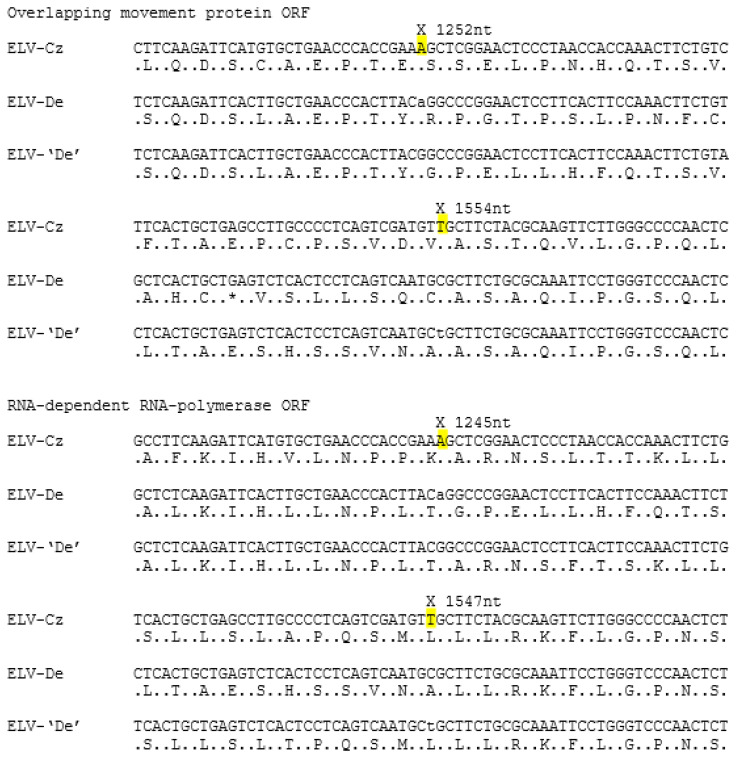
Changes that correct two possible indel errors in the published ELV-De sequence. For each of the regions involved, three sequences are shown—ELV-Cz together with ELV-De, as published, together with ELV-‘De’ after the changes. The nucleotides to be removed/added are given in lowercase. The positions of the proposed two changes are highlighted and shown (X), and their positions given for the gene involved (N.B. nt 1252 in the MP gene) are the same as nt 1245 in the RdRp gene.

The ELV-De MP gene is shorter (441 codons) than that of ELV-Cz, but an examination of the nt and encoded aa sequences of this region (Figure 2) showed evidence of a frameshift and the removal of nt 1252 of the ELV-De MP gene and insertion of an extra “T” at nt 1554 significantly increased homology between the ELV-Cz and ELV-De in this region and removed all termination codons. The resulting ELV-‘De’ MP is the same length as that of ELV-Cz, and the RdRp gene and its encoded amino acids were not significantly altered.

Analyses of ELV-Cz together with genomic sequences of other tymoviruses using the RDP program found no evidence of recombination, and maximum likelihood (ML) phylogenetic trees of individual genes, or their concatenate, confirmed that ELV-Cz and ELV-De are closely related isolates of the same virus, and are only distantly related to other tymoviruses, as reported in 1990 for the CP genes of only five tymoviruses [3]. Sequence analysis revealed that the ELV-Cz RdRp ORF had an 88.1% nt (92.7% aa) identity with that of ELV-De (NC_001977, AF098523) ORF (Table 1).

This report presents the near-complete genome of ELV-Cz and is the second isolate of ELV to have been sequenced; the earlier one is a German isolate. This analysis confirms the earlier report of ELV-De but identifies two single nucleotide changes in its MP/RdRp gene that may have been incorrect and which, if changed, increased the homology between ELV-De and ELV-Cz in this region [3,4]. It is notable that ELV-De was sequenced by the Sanger method, in contrast to the sequence ELV-Cz, and the subject of this report, which was obtained through HTS analysis and is used to identify single-nucleotide polymorphisms (SNPs) [8,9,10].

The current report contributes to our understanding of tymoviruses and the role of the MP gene in the spread [11] and symptoms [12] and in the interpretation of tymovirus phylogeny (in preparation).

## 3. Materials and Methods

### 3.1. High-Throughput Sequencing (HTS)

ELV was extracted from *Sisymbrium altissimum* leaves collected in the Czech city of České Budějovice in 1992. It was propagated in *Sinapis alba* plants, and the leaves were freeze-dried [6] and stored. Total RNA was extracted from stored dry *Sinapis alba* leaves using the RNeasy PowerPlant Kit (Qiagen, Hilden, Germany). The samples were treated with DNase I (Ambion, Austin, TX, USA). The ribosomal RNAs were removed using QIAseq FastSelect (Qiagen, Germany). The library for high-throughput sequencing (HTS) was prepared using the Illumina DNA Prep Kit and sequenced in a NextSeq2000 instrument (paired-end 2 × 150) at the Leibniz Institute DSMZ (Braunschweig, Germany). Bioinformatic analyses were achieved using Geneious Prime version 2023.2 (Biomatters, Auckland, New Zealand). The raw reads were trimmed, normalized, and filtered using sequences of *Sinapis alba* and assembled de novo. The resulting first 1000 contigs were subjected to BLASTn analysis against a custom database (viruses and viroids) in order to identify any such sequences, as described previously [13].

### 3.2. Genome Characterization

The NCBI ORFfinder was used to predict the corresponding open reading frames (ORFs) (https://www.ncbi.nlm.nih.gov/orffinder, accessed on 7 July 2024). The resulting viral sequence was compared to that of ELV-De obtained from Genbank using BioEdit 7.2.5 [14].

A maximum likelihood (ML) phylogenetic tree was constructed from the CP gene sequences of 10 tymoviruses, including the sequences of both ELV isolates, and using the Hasegawa–Kishino–Yano (HKY+G+I) model with 500 bootstrap replications, which was found to be the optimum by MEGA X [15].

A search for recombination events was achieved using RDP5 with default parameters [16].

## Figures and Tables

**Table 1:** Nucleotide (nt) and amino acid (aa) sequence identities (%) of the Czech (PP093825) and German (NC_001977) ELV isolates.

	RdRp		MP		CP	
Czech isolate vs. German isolate	nt	aa	nt	aa	nt	aa
	88.1	92.7	87.9	72.4	92.2	99

## Data Availability

The virus genomic sequence obtained in the present work was deposited in the GenBank database of the National Center for Biotechnology Information (NCBI) under accession number PP093825 (BioProject ID: PRJNA1099901). Further data that support the findings of this study are available from the corresponding author upon request.
